# Cottage Asylums

**Published:** 1861-04

**Authors:** W. A. F. Browne

**Affiliations:** one of the General Board of Commissioners in Lunacy for Scotland.


### Art. V.—COTTAGE ASYLUMS.

By W. A. F. Browne, one of the General Board of Commissioners in Lunacy for Scotland.

"En principe tout hospice, tout établissement fermé, est de son essence et par lui-même la négation du traitement naturel ; l'un est la prison, l'autre est la liberté." (p. 99.)—Duval : *Gheel, ou Une Colonie d'Aliénés vivant en famille et en liberté. Paris,* 1860.

"Ce n'est que dans un établissement central, que l'on appellerait infirmerie, que le traitement purement médical pourrait être administré ; tant que Gheel en sera privé il restera un établissement incomplet." (p. 35.)—*Thérapeutique Naturelle de la Folie. L'Air libre et la Vie de la Famille, dans la Commune de Gheel. Par Dr. J. Parigot. Bruxelles,* 1852.

Dr. Willers Jessen has written as follows:*—"Roller has repeatedly given his opinion against private, and in favour of public Asylums. He has not, it is true, given reasons for this preference, but should he continue to defend colonies for the insane, he must show how the reasons against private asylums and for colonies may be reconciled. Browne* has expressed another peculiar opinion. He adduces the following reasons against colonies :—Firstly, the incompatibility of such a plan with the general economy of villages and communities in Britain, with the tenure of property, and with the habits of the people. Secondly, the doubt whether the arrangement, if food, clothing, and medical attendance were provided, as in the asylums, would prove lucrative. And thirdly, the certainty that severity, cruelty, and neglect would arise when the responsibility is so small, the temptation to fraud and tyranny so great, and the chances of detection so few. An experiment," he continues, "of a somewhat similar kind failed in Arran. Even in asylums in which the superintendence is permanent, in which they have honest and respectable attendants, whose interest, as well as their character and ambition, are concerned in the prosperity, health, and love of their charges, there appear daily violations of duty and humanity ; and these offences are quite as frequent against the weak and peaceable as against the furious. Even the dwellings of the patients are converted, through harshness and filth, into unpleasant places of abode.

"The hopes of philanthropists rest upon the combination of the cottage system with that of a central asylum, where the families of the attendants would represent the peasants, or where peasants would become tenant servants within the grounds, under the rule of the Medical Governor and his staff. This is, at all events,

---

* "Ueber Irrenkolonen und andere Nothbehilfe der Krankenpflege."—*Allgemeine Zeitschrift für Psychiatrie,* August, 1859.

original, and so much the more worthy of consideration, because its application might remove an evil which no colony can otherwise escape. As is easily seen, the reception of insane patients must raise the value of the ground, as also the price of farming and rents in the colonies above their worth in the neighbouring parishes. Only the original possessors, and perhaps the first purchasers will, therefore, receive the full profit from the board of the insane, &c. For the execution of the plan the asylum must, of course, possess a considerable area; but not of necessity the funds to raise buildings, for under suitable conditions, every colonist might be made to furnish money for the building of a dwelling. The buildings themselves would be erected and placed strictly upon medical principles, and sites would only be granted to proved trustworthy persons. All this appears very excellent, and gives an undeniable superiority to Browne's plan over colonies formed upon the discouraging model of Gheel."

To this article Dr. Mundy, the author of various pamphlets upon the same subject, such as "*Gheel est un Asile Patronal,*" "*L'indifférence de notre siècle pour l'infortune des Aliénés,*" has given an energetic reply in "*L'institution des colonies d'Aliénés; Gheel et ses Adversaires.*" As, however, Dr. Mundy conceives that my proposal is a recommendation of Gheel, as he concurs in the propriety of rendering the hosts, or guardians, responsible paid servants; and, as he does not participate in the extreme views of Dr. Parigot, it will not be necessary to allude further to his labours.

There appeared in *The Journal of Psychological Medicine* for July, 1860, and in the *Revue Trimestrielle,* vol. xxvii., an article on the "Reform of Lunatic Asylums," by J. Parigot M.D., Inspector of Lunatic Asylums for the Arrondissement of Brussels, &c., in which these views are thus commented upon:—

"It appears (still following the remarks of Dr. Jessen, of Hornheim, near Kiel), that Dr. Browne, Inspector of Lunatic Asylums in Scotland, has made certain observations unfavourable to colonies. As Dr. Jessen reproduces these objections, we will answer the first one, to wit, that the financial administration of a village, subject to feudal rights, would prevent the establishment of a colony, by observing that Government in this country can buy up these feudal rights without injuring any one's interest, but, on the contrary, to the advancement of every one's interest, if a colony be deemed useful. As to the second objection, that, after all the expenses incurred for the keep and clothing of the patients, as in asylums, there would be no profit, we would reply, that this is a great mistake; for, admitting even that the expenditure was the same (which is not the case, as we have shown), there would remain a double number of cures to the credit of free air, conjoined with the rejection of useless discomforts for the incurables." (p. 293.)

Since the publication of this paper, the author has visited this country, chiefly to preach a bloodless and philanthropic crusade in favour of his own creed and against the prison-asylums, as he stigmatises them, which exist here; and I have had an opportunity of cultivating his friendship and of forming a high estimate of his benevolence, enthusiasm, and ability. It is expedient to express thus explicitly and strongly the respect entertained towards Dr. Parigot, and the confidence placed in the sincerity with which he endeavours to propagate a new system of treatment; because it will become necessary to characterize that system, and especially the mode in which it is advocated, in less approbatory terms. While fully appreciating the romantic interest, the historic prestige, the psychological curiosity, the substantial and, above all, the suggestive elements of good connected with the community of Gheel—it is foreign to the purpose and spirit of these observations to denounce, as M. Parigot has done, those who fail to arrive at the same conclusions, or who regard them as erroneous, as "speculators and traffickers" in madness (p. 280), whose "arguments depend upon their interests and their prejudices" (p. 294), "who may seek to retain captive unfortunate beings whom, most frequently, unnatural relatives, from disgraceful motives, wish to get rid of" (p. 280). Such a class I do not believe to exist in this country; nor can I credit the assertion, "that passers-by are imposed upon," or that it is the intention of any one "to impose upon passers-by by the sound of music, dancing, fêtes, and entertainments of various kinds, while the desire to cure is either altogether absent or lightly appreciated." These rash accusations and the insinuations conveyed in the terms, "mercantile idea," "contempt of science," "personal interest," and the bitterness of feeling from which they appear to emanate, and which they are undoubtedly calculated to produce, shall be placed out of view, and an attempt made to discuss the "Traitement à l'air libre," &c., as a curative agent, or as a means of removing difficulties known to exist, and known greatly to embarrass and impede the exertions of men of science as well as of the philanthropist, in so far, at least, as these considerations are involved in, or affected by, the proposal made by the writer and commented upon by Drs. Jessen and Parigot.

As this suggestion has assumed, in the hands of such commentators and critics, somewhat the aspect of a discovery, and as it has become probable that establishments for the care of the insane may be constructed in accordance with this view, as the form in which it originally appeared must be unknown to your readers, and especially as that form has been somewhat distorted and shorn of its fair proportions in the attempts to illustrate or demolish, it may be prudent to introduce the following extract from page 8

of the *Eighteenth Annual Report of the Crichton Royal Institution for Lunatics, Dumfries.* It must, however, be premised that the existence of Gheel for centuries is not, as Roller* argues, a proof of its rationality, but merely of the practicability of retaining lunatics in one locality, partly by force, partly by superstition, partly by kind and indulgent management. The Gheel of a thousand years exists no longer. This is no protest against a modified form of the community. The humane impulse which has changed the condition of the lunatic in many lands was late in reaching West Flanders. The Gheel painted by M. Parigot, is of yesterday; it is inchoate, in progress and in the act of development towards that alteration now proposed.

"The moral evils of a vast assemblage of incurable cases in one building are greater still. The community becomes unwieldy; the cares are beyond the capacity of the medical officers; personal intimacy is impossible; recent cases are lost, and overlooked in the mass; and the patients are treated in groups and classes. An unhealthy moral atmosphere is created; a mental epidemic arises, where delusion, and debility, and extravagance are propagated from individual to individual, and the intellect is dwarfed and enfeebled by monotony, routine, and subjection. So pressing have these evils become, that remedies are anxiously looked for. The construction of smaller establishments increases the expense of maintenance so much, that additions to the staff of officers, the subdivision and classification of the inmates into distinct bodies, and the erection of separate buildings dependent upon a central administration and economy, and the introduction of the system resorted to in Belgium, of placing fatuous and tractable cases in the houses of respectable peasants, but under constant medical superintendence, and where their labour is received in payment of maintenance, have all been pointed to, as fully or partially meeting the difficulties. The objection to the scheme of erecting colonies for the insane, such as exists at Gheel, which was originally founded on religious views, subsequently persevered in for economic ends, and has latterly been placed under an enlightened policy, are first, the incompatibility of such a plan with the general economy of villages or parishes in Britain, with the tenure of property, and with the habits of the people; secondly, the doubt whether the arrangement, if diet, clothing, and medical attendance were supplied, as in asylums, would prove remunerative; and thirdly, the certainty that hardship, cruelty, and neglect would spring up, where the responsibility was so slight, the temptations to peculation and tyranny so many, and the chances of detection so few. An experiment of a somewhat similar kind in Arran terminated in failure. Even in asylums where the supervision is sleepless, where there are trained and respectable attendants, whose most selfish interests, as well as their character and ambition, are involved in the

* *Roller,* Lib. cit.

well-being, health, and love of their charges, violations of duty and humanity daily occur; and these derelictions are as frequent where the imbecile and gentle are concerned as the furious. Even the homes of the patients are converted into prisons and shambles, from positive callousness, or sordid selfishness. The hopes of philanthropists rest upon the combination of the cottage system with that of a central asylum, where the families of the attendants would represent the peasants, or where peasants would become tenant-servants within the grounds, under the rule and direction of the medical governor and his staff."

It will be observed that the merits eulogised by Dr. Jessen are rather inferences, or may be made results, from the application of the plan, than characteristic of its nature; for, although economic views are not disregarded, they occupied a very subordinate rank in the estimate of the writer. It will be further apparent that Dr. Parigot addresses himself to answer two only of the objections urged to the introduction of such a colony as Gheel into Britain, and that he has misunderstood these. The words, "feudal rights," do not occur in the passage cited; they never occurred to the thoughts of the writer. The "tenure of property," referred not to "feudal rights," but to the difficulty which must be encountered in a densely peopled country in acquiring a territory such as would be necessary for the purpose, and to the necessity for creating an agricultural colony previous to its conversion into a colony for the insane. The word "remunerative," was not intended to imply "profit," for such an element cannot enter into a consideration of the management of the affairs of the pauper lunatic, but "loss;" and it is still, in my opinion, extremely doubtful whether the number of cures be such as to justify perseverance in an experiment conducted as it appears to have been previous to 1856. Esquirol, upon the authority of Dr. Backel, who passed his life in Gheel, states, in 1821, that there were from 10 to 15 recoveries per annum in 400 or 500 patients. M. Duval, writing in 1860, represents the average number of recoveries as having been 36 in 900 patients, for four years. M. Bulckens, Medical Inspector of the colony, furnishes, in his Report to the Belgian Commissioners in Lunacy, 1856, a table which shows, that of a gross population of 765 lunatics, of whom 127 had been admitted during the year, 29 had been discharged cured, 10 improved, 32 unchanged, and that 6 had escaped, and 63 died.

In investigating the subject more in detail, it will be vain to accumulate a large mass of authorities upon the main points at issue, for Gheel has now a voluminous literature; but it will be expedient to adduce some of the evidence, and that chronologically, which seemed to lay bare the inherent weakness of the principles upon which the colony was originally conducted, and upon the detection of which that project, now about to be realized, was founded. From a copious account of a visit paid by Esquirol to Gheel, in 1821, the following extracts will suffice:—

"The insane sent to Gheel generally labour under chronic, or incurable forms of derangement. It must not be supposed that the streets and fields are crowded with lunatics. I encountered a small number. The females do not go out much. If excited at home, their violence is speedily repressed. About 50 males are employed in farm works. The board ranges from 200 to 1200 francs. Those who reside in the village are much better attended to than those who are entrusted to the rustics. I have seen some who were comfortably accommodated, but the majority, *le plus grand nombre*, are very badly kept. No doubt can be entertained but that a higher degree of utility could be given to this singular establishment. I had the honour to propose to the Minister of the Interior (Holland) to erect an asylum which could receive such lunatics as, by their agitation, their violence, and filthy habits, are most exposed to bad treatment from their hosts; while the peaceable and cleanly might remain in private dwellings. The medical superintendent and his staff would, at the same time, be called upon to exercise a constant supervision over the isolated lunatics, and over the conduct of those to whom they are confided."*

Stimulated by the narrative of my distinguished and much-loved preceptor, by a desire to examine so curious and ancient a community from a psychological point of view, and actuated, perhaps, by the spirit of adventure—for the spot was then a remote village in the middle of a sandy waste—I visited Gheel in July, 1838; and it may be considered a sort of distinction, I was the first medical man from this country who had penetrated the district. From the notes then taken some portions shall be given:—

"There are no gentlemen's houses in the district; and the farm-houses, though neat, and generally surrounded by trees and a garden, are evidently in the hands of the poor. Even their frequency shows this. They are sometimes built of brick, but much more frequently they are constructed of wattled, or wicker-work, laid thickly over with mud or plaster, and whitewashed. The roof is large, deep, and thatched. Such are the residences of the peasants who inhabit the more distant, as well as the urban part of the commune. The interior consists of two rooms in front, and some sleeping cabins behind, opening upon a court. The apartments are large, paved with square bricks, having an enormous fireplace, above which is invariably a row of plates, generally of pewter. The houses are generally dirty and confused. The people

---

* *Notice sur le Village de Gheel, Maladies Mentales,* Tom. ii. p. 715-20, 21, &c.

keeping them seem to be about the rank of English cottagers, but are inferior in aspect, tone of character, and cleanliness of habits. The charge is almost invariably from 180 to 200 francs per annum. When they are admitted to the conventual building something additional is paid. For this sum they are lodged, fed, and supplied with bedclothes. Their body clothes are furnished by the commune to which they belong, or by their relatives. They, of course, eat with the family, who generally subsist upon bread, milk, and vegetables. I saw many of them at their meals. One had a mess of greens and potatoes, another potatoes fried in milk, a third potatoes boiled in milk, and a fourth was eating bread, but demanding tobacco. I visited the convent for the *better* treatment of the more difficult and violent cases. This is the only case in which it is freely acknowledged any treatment was attempted. It was hinted that the said treatment was of a religious character; but it is difficult to conceive what is meant by this, except a solution be found in the proximity of the house to the Church of St. Dymphna. The place is horrible—old, dark, dirty. The rooms in which the patients are placed are mere dens cut in the wall, with a window opening into the large common room. At the side of the fire-place are enormous rings and chains, which are intended to restrain patients when troublesome, and in winter. The impression produced by this awful scene and the coarse female who superintends the imposition of this restraint was distressing. I visited many of the insane in their cottages. The majority of those I saw in town were in bed; some of them bound to it by chains, and, with one exception, in the most disgusting state of filth and degradation. The smell and aspect of the dens in which they lay were intolerable. One man, formerly an officer of Lancers, tall, strong, hairy, will not get up, yet he was chained to bed. He passes everything where he lies, spits all round, howls night and day. His bed was of tan, or oak bark. The place was dark and loathsome; the whole house filthy. My informants said that about one-third are chained or strapped, to prevent them from striking or escaping. Many are employed in various ways, but especially on the farms and gardens of those with whom they reside. Considering that the houses visited in the country were worse, the lodgment of these unfortunates was better, but still very bad. They seem to entertain an affection for their keepers; and one, an idiot, expressed great fears that we had come to remove him from his mother. There was exhibited in one of these rural establishments the iron girdle used to confine the refractory. We met many of the lunatics wandering about in the lanes, some of them hobbled, confined, some of them free. All classes seem to depend upon this traffic. The burgomaster had a farm in the country, which I visited, and found there at least two insane boarders. There exists a commission, appointed by the town, and consisting of two medical men and of some of the respectable citizens, who examine into the condition of the boarders, and see that they are properly attended to. If this be not the case, the patient is transferred to another house. This is regarded as a great punishment by the keepers, as they depend greatly on the board. An institution for the insane has been suppressed, and the patients, 30 in number, sent to Gheel. Why is this?"

Apparently, in answer to such an interrogatory, my friend and fellow-labourer, the celebrated M. Guislain, wrote during the same year, 1838 :—" Gheel is a locality to which lunatics are often sent, on account of its cheapness. It is a sort of colony of lunatics, which, from the singularity of its aspect, has been an object of admiration, in which I do not participate."* It is understood that, towards the close of his life, M. Guislain adopted a more favourable opinion of the capabilities of Gheel.

M. Brierre de Boismont's examination of Gheel took place in 1846, and is recorded in a most valuable contribution to the *Annales Médico-Psychologiques*, vol. iv., Second Series, October, 1852, as a review of M. Parigot's *Thérapeutique Naturelle de la Folie.* It is only necessary to quote the following paragraph :—

" There is no treatment of alienation. I learned that the patients are never opposed. The dirty were a special object of attention. I found many of them seated and fixed upon a chair with a pierced bottom. I saw one blind idiot of excessively dirty habits; but, such was the care of his nurse, that he had no smell. The excited and destructive wore camisoles. I found many melancholics in bed. It is, without doubt, the way to perpetuate their malady. Nothing has been done for the physical or moral treatment of the patients. There are no baths, no infirmary for acute cases or incidental diseases. Elopers, mischievous, homicides, incendiaries, kleptomaniacs are chained. I entered about twenty houses. They were clean, resembling those of our peasants in furniture, &c.; and, though bare, were clean and tidy. The clothes were clean; the food that of the hosts. I would preserve Gheel, but solely as an establishment for the incurable."

Discussions took place on the pretensions of Gheel in the Parisian Medico-Psychological Society, in June and July, 1860.† Their most marked characteristics were denunciations, from some honoured lips, of everything that was not French, and the appointment of a committee of inquiry.

My friends Dr. Webster visited Gheel in 1856, Dr. Stevens and Dr. Coxe in 1857, and Dr. Mitchell and Dr. Sibbald in 1860; and I have read the various reports in which they have recorded the impressions received, but I have purposely refrained from availing myself of the important information which these documents supply, as I was desirous that my personal observations, as well as my opinions, should be contrasted and tested by competent authorities speaking the same language, holding the same faith, accustomed to the same habits and manners as the writers

* *Exposé sur l'état actuel des Aliénés en Belgique*, p. 11, 1838.
† *Ann. Médico-Psych.* Janvier, 1861, p. 107, &c.

who support the principle of insane colonies in its integrity, and, in an especial manner, by the distinguished physicians who have lived in the town, and are intimately acquainted with its condition and that of its inhabitants. Dr. Webster alone, it may be right to mention, espouses the cause of Gheel as it is; denounces the proposal of breaking up and discontinuing the colony as "an act of sheer vandalism; insane colonies should rather be established elsewhere, and thereby take advantage of former practical knowledge, based upon the long experience thus obtained. Other countries might even advantageously imitate the example thus furnished."*

This ascending series of selections is not given in order to depreciate the colony of Gheel; nor with any conception that it will lessen the wonder and admiration which it is calculated to excite; but to indicate the influences by which my own thoughts were directed *from* what existed *to* a future development, embracing some of the peculiarities by which it is marked; but likewise another and important element, that of an *Hospital.* The attempt was incumbent to endeavour to separate the astonishment and curiosity suggested by a community differing so essentially from all others, of which a considerable portion of the citizens were insane, at liberty and not amenable to law; which had subsisted for perhaps a thousand years, and which had been kept together by a tradition;—from its characteristics as an hospital for the cure of disease, in virtue of these very peculiarities, and by the operation of religious faith. The result, or residuum, of this analysis was to present the "ruins of living men," of all ranks and positions associated promiscuously with bluff and burly peasants, enjoying an amount of liberty still unknown, and of license still happily unheard of elsewhere, imperfectly superintended and apparently no longer under, or very indirectly under, the care of priests or physicians. It afforded, in fact, the last glimpse of a mediæval condition, incrusted with the stains and decay and corruption of a worn-out organization, where the faith in the supernatural had faded away, and the sun of science had not yet arisen. There was forced upon the attention these considerations:—

I. That there was and is no presiding and supreme medical authority, interpenetrating and overruling every department and detail, such as is required and eminently useful where bodies of men are individualized and deprived of a common purpose by insanity or crime. With great respect towards Dr. Bulckens, the free air system, as represented at Gheel, is medical nullifidianism, and inevitably entails the relinquishment of every, or many, other means of alleviation as omnipotent as fresh air. It is metonomy

* *Journal of Psychological Medicine*, vol. x. p. 235.

to designate the turning the insane adrift into the fields, or the most unlimited indulgence in the open air, a remedy. It is a condition of general health, it may enter into curative measures; but not more in insanity than in other disease. If itself remedial, lunatics at home or roaming over our mountains, as they still do in great numbers, would be most advantageously situate for recovery. It is not necessary to advert to the 871 pharmaceutical prescriptions issued to 765 patients in Gheel during the year 1856* farther than to illustrate the impossibility of treating insanity under the circumstances described, and the encouragement given to the heresy of trusting to moral aid exclusively.

The various calls upon the time and thought of the medical superintendent of a large asylum render the daily or frequent visits to his patients difficult and laborious. How such duties can be performed when nearly a thousand lunatics reside in distinct houses, scattered over a "commune qui n'a pas moins de neuf lieues de périmètre," and are generally engaged in the fields; even when the medical staff consists of a physician and four assistants —is not easily understood. Such visits in an asylum require frequently to be of long duration, and have for object to afford professional aid either to physical, or moral, or imaginary suffering;—they may be to determine progress, to inquire into the deportment of subordinate agents, the state of the dress, the apartments;—or to soothe and sympathise with, to plant a hope, or to uproot an error; but in the case of a colony where the patients may be far removed from inspection and redress, where they are servants as well as boarders, and may be treated as slaves; where they are subjected to the dominion or guidance of a whole family, and where a contract exists involving not merely their maintenance but their productive capacity and degree of usefulness and degradation; for a larger allowance is given for the dirty and demented than for the well-conducted and industrious,† and, by a strange miscalculation of the interests of a particular lunatic, a guardian is punished for negligence or failure in one case by imposing upon him another still more unmanageable and unprofitable;—these inquiries assume a much more grave and important and complicated character, in addition to the ordinary objects in view. The opinion of M. Parigot that a weekly visit‡ may suffice for any case is so inconsistent with the purposes in view, and with the conception of a lunatic under treatment requiring separation from his friends and extradition to the more remote farms in Gheel,—as to countenance the impression that a central building already exists, where acute and urgent cases are

---

* Bulckens, p. 39.

‡ Parigot, p. 96.

deposited. The value of the supervision and of the moral influence established by constant medical attendance is such as to constitute a strong argument in support of what may be called the composite plan.

The scattered population of Gheel places it very much in the same category as those vast asylums in this country, where the physician has to traverse miles of passages and ascend staircases of mountain altitude; where an acquaintance with the mind and dispositions, and even with the name and features of each patient, is next to impossible; where the relation of friend and monitor is lost in that of director, and the knowledge of the very existence of the phenomena of disease must depend upon the reports of stupid, stolid, ignorant, and perhaps, unwilling witnesses. The detection and management of the various forms of insanity under such circumstances appears a problem to those who, like Bulckens,* have experienced the difficulty of making nurses understand that their charges are ill, and of teaching them, even when not allied to Mrs. Gamp, and in spite of Curwen's pharmacopœia, the commonest offices of the sick-room. And lastly, how the approaches of the abstinence so frequent as a symptom of melancholia and other forms of alienation, can be observed and watched amid such penury and ignorance, and when observed how counteracted, is perplexing. This is a momentous consideration in a Roman Catholic country, where fasting is a recognised expression of piety, and consequently, so frequently a symptom of the exaltation and exaggeration of the sentiment in disease.

II. There was and is no adequate guarantee for the humane or judicious care and management of the insane, or such as may be established under other circumstances, nor is there any advantage gained compensating for its absence. "All is to be attained by kindness, not by intimidation or violence." "At Gheel what is admired is the devotion and disinterestedness of the keepers."† Duval paints the population of Gheel as endowed with "bonté naturelle poussée jusqu'à l'extrême limite, calme du caractère comme de la démarche, imperturbable patience, en toute occasion un faire tranquille et mesuré, que le délire le plus aigu d'un aliéné ne parvient pas à troubler."‡ Parigot deplores that it has been impossible to reward "l'abnégation angélique de bien des nourriciers."§ Even the less ardent Bulckens declares "les nourriciers s'acquittent en général de leur mission difficile et souvent périlleuse d'une manière qui ne mérite que des éloges;" and he sums up these encomiums by these emphatic words: "It is

---

* Lib. cit., p. 26. † *Journal of Psychological Medicine*, p. 293, vol. xiii.
‡ *Gheel une Colonie d'Aliénés*, p. 67.
§ *Thérapeutique Naturelle de la Folie*, p. 119.

requisite to have a mission, which can only be inspired by religion, or be innate, as in the inhabitants of Gheel. To many these every-day cares, anxieties, and agitation have become a moral necessity of their existence."*

I would most reluctantly impugn the self-sacrifice and self-control and "love casting out fear" of these virtuous peasantry, their nation's pride; in fact, I am disposed to regard them as possessed of many excellent qualities. It is refreshing to find an Auburn, a Utopia somewhere, especially among the sand wastes of Flanders: it is not even necessary to quote the few instances of neglect and garotting recorded by Parigot,† of abuses alluded to by Bulckens, "l'abandon, un trafic honteux,"‡ or the regulations now existing, in order to prove that man is fallible, as I have to deal less with the abstract practicability of such an institution, or with its practicability in another country, than with the propriety of introducing it into our own. It would not even affect the argument to admit the impeccability of the Flemings; but to those who have lived for a quarter of a century among the insane, who have expended every energy in selecting, resorted to every expedient in instructing, training, rewarding suitable custodiers, the statement that 548 families, or more than 2000 individuals, can be found, "devoted and disinterested," by hazard, or having no other recommendation, and affording no other guarantee than residence in a certain locality, and that in some cases their ancestors were engaged in a similar guardianship; and that under the shadowy superintendence described, they, or a vast majority of them, continue to discharge their very sacred and difficult duties in a satisfactory manner, appears marvellous and delightful, and is assuredly altogether inconsistent with the experience of the medical governors of asylums. No one can be more alive to the trying position, to the rectitude, the forbearance, and the intelligence of many of the guardians of the insane than the writer; but he cannot forget, that, in order to secure the co-operation of individuals possessing such qualities, or some of them, hundreds of candidates must be tested and found wanting, failing even in the vulgar attributes of vigilance and attention; and that even those who occupy a higher place, who may be neither negligent nor cruel, rarely attain to a conception of "devotion and disinterestedness;" and are preserved in their status by sustained discipline. It must be farther noticed that the discovery and development of such qualities as entitle to trust, as well as the detection and punishment of unworthiness, take place under a system of daily, hourly, paternal superintendence, of constant and anxious scrutiny; and this within a compass and under cir-

* *Rapport*, 1856, p. 34. † pp. 78, 79. ‡ p. 5.

cumstances which secure a knowledge of individual disposition and capacity and a scope for instruction not otherwise easily attainable. This rigid and far-stretching or inquisitorial arrangement is founded less upon low views of human probity and sympathy with suffering, than upon what is believed to be a correct estimate of the frightful ordeal to which the highest natures may be exposed; of the provocations, the danger, the exhausting suspicion, irritation, ingratitude, which must be encountered, and under which even the ties of affection, lifelong associations, and selfish interests so often succumb. The signal failure and inefficiency of this class of officials have led many physicians to crave a total reorganization of this department of service, and to propose to incorporate some modification of the plan of enlisting the religious orders in the management of the insane, with our existing economy in asylums; or, at all events, to resort to some plan which may secure assistants actuated by higher motives than their subsistence, and from other classes than the idle, the illiterate, and the refuse of other trades, which at present generally supply them. Duval demurs to the employment of sisters of charity, as they cannot possess the "hereditary merits, &c. of the inhabitants of a country devoted to the treatment of such maladies" (p. 84).

It may be that confinement, monotony, that close constant association with unhealthy and debased minds, act detrimentally upon the disposition of those who are imperfectly constituted and educated, and tend to produce that indifference, hardness, harshness, and enfeebled conscientiousness which so often frustrate the hopes and measures of the physician. A similar morbid and malign influence must, however, if it exist at all, be diffused through the homesteads of the yeomen of Gheel by the constant presence of the insane inmates. It must present itself in even a more insidious and intense form. The exposure of the attendant to the infection is limited to hours. He escapes to his family, his home, his holiday. He spends his vacation in sleep, or amid healthful and invigorating impressions. But the skeleton, the demon of disease, haunts the Gheeloise hut for ever. It is a part not merely of household arrangements for good or for evil; it is a part of the inmost thoughts. In many examples recorded, and in thousands of others that literally waste their moral fragrance on that desert air, the imbecile is a child, a companion, a joy, a source of wealth and food; but he must still be a care, an anxiety, a pain; his presence cannot, in the majority of instances, regulate the passions, elevate the intellect, calm the temper. Nay, the same observation, to a certain extent, applies to those intrusted with the insane in their own houses; to the parents, relations, natural guardians. In one sense every parish in Scotland is an extemporised Gheel.

In one of these, possessed of the legal organization of a parochial board, inspector of poor, and medical officer, there very recently prevailed the following system of treatment, or precaution for the safety of the public :—Of twenty-two individuals labouring under different forms of mental disease, two were not seen; one was in bed; of six, the liberty did not seem to be interfered with; the subjection of one was secured by threats of a stick; two were confined to the house; one was shut, another locked into their bedrooms, when it was conceived necessary; one was manacled and shut into a box-bed; one was struck with the hand; and six were struck with what were called switches, but one of these assumed the proportions of a full-grown bludgeon in the eyes of those unaccustomed to such heroic measures. There was, possibly, no intentional cruelty in all this; but although resorted to as salutary or necessary discipline, it was undeniably the result of gross ignorance, irresponsible power, and domestic tyranny.

The jealousy of the public among us is keenly alive to the possibility that an educated man, enjoying a reputation for honour and integrity, and occupying a position which depends upon the possession of such qualities, may be blinded to the less palpable forms of mental disease and to the faint dawnings of convalescence, by interest in the prolongation of illness in an affluent charge: but to what extent would suspicion be justifiable in the case of the 584 nurses and their families, who are ignorant, indigent, and depend for sheer subsistence upon the board and the labour of their lunatic servants, may be inferred from the observations of M. Parigot (p. 17), as to the odious traffic in Lunatics, and from the Regulations* for 1838 containing prohibitions against bribes; authorisations for the imposition of restraint during excitement, or to prevent escape; declarations of "infamy" against those who strike a lunatic, except in self-defence; small fines on the occurrence of suicide; accidental death from negligence; on the discovery of dirt, ecchymoses, or gangrene!

III. That "Ne pas contrarier l'aliéné, lui permettre même toutes ses fantaisies tant qu'il n'y a dommage, ne lui rien imposer de force, tout obtenir par l'attrait, cette est la science suprême du gouvernement des fous á Gheel," is very dangerous, as well as false philosophy. In such phrases as "nothing opposes him," "he does with his time just as he pleases," used by Parigot, appear indications of an erroneous view at once of human nature, the very framework of sane society, and of the moral treatment of those of unsound mind. The condition of the greatest and most

---

* *v.* Parigot, Duvac.

independent intellects is imperfect, if they have been opposed in nothing. Education consists in a series of restrictions, concessions, sacrifices to the will and interests of others; society is kept together by antagonism, as well as by concurrence, by the absorption of the individual will, wishes, tendencies in common interests and before laws, authority, conventionalities; and lastly, the uprooting of irrational opinions and modes of action, the opposition to excited passions and propensities, the exposure of delusions, and even the subjugation of capricious and irregular habits to order and punctuality accomplished directly or indirectly, as may be required, constitute more effective means for the cure or mitigation of insanity, than a course which affords gratification through indulgence and non-interference, and purchases quiet at the expense of perpetuating disease. That a remedy is of doubtful efficacy or effect, or to be discarded because it is painful or repugnant to the patient, is as unsound doctrine in the treatment of psychical as of physical diseases. It sometimes happens that the pain and the repugnance are in themselves remedial; that actual cautery has roused attention, or diverted from mental sorrow and suffering to the irritated skin. Believing that the impression produced upon the wandering and disorderly and rebellious mind by the known existence of discipline, and of a mild but majestic authority, such as pervade our asylums, even where their requirements are scarcely felt; and by the obedience of the propensities, and peculiarities, and pursuits to guidance and government, and by their fusion into a general movement, are eminently beneficial, it is impossible to regard with complacency the negation of such influences, and of *all* substitutes for them, and the virtual abandonment of one of the most important means of moral treatment at Gheel, and the delivery of the diseased over to their own impulses, or to those of the good-natured but unenlightened persons to whom they are intrusted, often inferior to their charges in capacity, energy, and education, and who, in many cases, do not speak the same language, and cannot communicate advice or admonition were they so disposed. M. Bulckens, generally so modest and free from partisanship, in making the confession "on a objecté contre l'établissement de Gheel que la séquestration des aliénés dans une localité où on ne parle pas leur langue,"* actually enters upon a lengthened series of propositions to demonstrate that, far from being unfavourable to happiness and recovery, this isolation presents incontestible advantages!

IV. The amount of restraint is painful and unjustifiable. Much of it is permanent; and in an expurgated body of lunatics, which

* *Rapport*, p. 35.

is not only originally selected on the ground of the tractability and gentleness and perhaps of the incurability of its members; but from which individuals, homicides for example, are from time to time withdrawn as they become unmanageable,* and out of which the impatient and recusant remove themselves by evasion,† it must be attributed to the timidity of the custodiers, a quality almost incompatible with kind management. Again, it is resorted to, not even upon the pretext of treatment, or to facilitate the application of treatment, but upon the bald, broad, intelligible reason of coercion, to repress, to confine. During 1856, it is ascertained from a table, prepared by M. Bulckens,‡ that eight patients had worn the camisole, sixty-five ankle-chains, twelve the iron girdle with wrist-chains, and eight the iron girdle with ankle-chains; in all ninety-three. Dr. Mundy saw some leg-chains, but no camisole nor strong chair, in January, 1860. Dr. Sibbald, in the spring of the same year, encountered about thirty patients confined by manacles or some "more gentle means of restraint." It does not enter into the speculations of the extreme defenders of the "air libre," that personal liberty and even the gesticulations which are resorted to under restraint, may be prejudicial; that the economization of strength and tissue by some means was considered a justification of the use of the strait jacket and padded rooms; that exercise is interdicted in mental diseases of cardiac origin; that one object of seclusion in an asylum was to remove from noxious and disturbing influences, to obtain rest and quiet; another, to render treatment of any kind practicable; a third, to protect the sufferers from evil; and a fourth, to minimise or abolish personal restraint; or lastly, that the most eminent psychologists, though repudiating the notion that any mysterious or therapeutical influence is attributable to the mural or other inclosures around an asylum, or to its size, are well assured by experience that discipline and treatment can be better applied in one particular form of a house than in another, and, to a certain number of inmates. In place of there being an antithesis, as M. Parigot says (p. 285, *Psych. Journal*), in giving liberty to a number of lunatics forcibly kept together within restricted limits, Gheel, and the restraint used there, may be adduced in illustration of the proposition that, as a consequence of structural arrangement in a central building, personal liberty becomes practicable and safe.

A Frenchman, it may be recollected, at one time recommended that strait-jackets should be made of velvet for the patricians, and that muffs should be decorated with ribbons in order to render

* Dr. Webster, vol. x., *Journal of Psychological Medicine*, p. 220.
† B. de Boismont, op. cit. p. 531. ‡ *Rapport*, p. 32.

them less humiliating. I do not know that golden chains or crinoline strong dresses ever came into fashion; but it is certain that the publicity with which the badge is worn, M. B. De Boismont first heard the clank of chains during a religious procession; and the reasons for which it is known to be worn, must brutalise both the wearer and the spectator. Apart from this consideration, it is, perhaps, better that the iron should enter into the soul in the open air than in a vitiated atmosphere.

V. That there does not appear to be such an amount of employment as to distinguish the community from other large bodies of the insane differently situate, and as is claimed as a distinction; and, as might be expected, where there is not merely an interest, but a stern necessity, to tax the physical powers of the industrious to the utmost; where there is an investment in muscular force or dexterity, and labour becomes the payment of an obligation rather than a cure for madness. There is, in the fourth article of the special order of 1851, a provision against tyranny and exaction of this kind; but how such an offence can be detected it is difficult to understand. M. Parigot sneers at classification, as serving but to "render life more endurable to the prisoner."* The endurability of life is an important mode of cure or amelioration. It may be a matter for inquiry whether the presence of a dirty element would promote or impede the recovery of a melancholic or a monomaniac; but there can be no doubt as to the propriety of that grand step in classification, which "deprived the colony of all lunatics suspected, on whatever grounds, to have suicidal, homicidal, or dangerous propensities" (p. 284, O. C.), a measure which, in conjunction with the segregation of the patients actually sent, renders Gheel itself the most striking example of classification carried, I do not assert to extravagance, but as far as it could go. By a very inartificial but perilous arrangement, and for obvious reasons, the colony is divided into three cordons or circles. The docile reside in the town; the more excited, amounting to 263, in suburban cottages; and the agitated and turbulent, 34 in number, are placed in the most distant and inaccessible hamlets, which are identified with seclusion-cells, and where the exiles may shout and wander unrestrainedly among the woods and wilds. A classification of the persons authorised to receive the insane has been attempted; but it is founded chiefly upon economic considerations and irrespective of mental qualifications, except in so far as these may incidentally be comprehended in regulations concerning moral deportment, attention to cleanliness, sufficiency of food, and the salubrity of the site of the house. It consists, 1. Of those who

* *Journal of Psychological Medicine*, vol. xiii. p. 283.

possess houses of their own. 2. Those who are tenants of the houses which they occupy. 3. Those who are the proprietors of the farms which they improve by the aid of their boarders; and, 4. Farmers in the country. The board of all the paupers is the same, but an additional sum is allowed where the habits are degraded. It would appear, however, that a robust and active labourer or handicraftsman is coveted as a good bargain, that a species of competition has been detected in the attempts to secure such a charge, and that bribery and corruption have been resorted to in prosecution of this end.

M. B. de Boismont reports that on the 1st October, 1840, of 681 individuals, of whom 324 were men and 357 women, 372 were engaged in occupation of some description, 309 were unable or refused to work, and that, in addition to these, 36 men and 13 women were in seclusion or under restraint, and consequently prevented from working; making a total of 358 who did nothing.*

He lauds the products of their industry. It may be interesting to compare with these observations the results in two British asylums, of nearly the same amount of population as Gheel, and of which a large majority labour under dementia, where agricultural labourers must be in a minority, and where labour is not inculcated as a panacea. In Hanwell, during 1856, of 507 men, 250 were employed, 114 in open air, 52 in galleries, &c.; of 657 women, 388 were employed, 20 in open air, 160 in wards, 186 in needlework. In Colney Hatch, during the same year, of 546 men, 246 were employed, 69 in open air, 80 in galleries; as upholsterers, 13; as carpenters, 11. Of 748 women, 503 were employed; in galleries, 125; in laundry, 72.

M. Parigot offers objections to work performed in an asylum; first, because it is compulsory; and secondly, because it is not in the open air.† There is, however, another paragraph by M. Parigot, which considerably modifies the force of the first argument. "La première violence morale surmontée," he says; "l'aliéné s'etant soumis (quelle que soit sa repugnance a s'occuper de travaux qu'il croit de lui) la diversion s'opere," &c.‡ But why should labour be more voluntary in a colony which a man cannot leave in consequence of iron cinctures, straps, &c., than in a confraternity from which his escape is prevented by walls; when in both it is the means of distraction, enjoyment, the price of additional indulgence or confidence, the proof of returning sanity, the condition upon which liberation depends. As to the second objection, it is very doubtful what would be the physical effect of compelling the population of heated manufactories, the

---

* Op. cit. p. 529. † *Journal of Psychological Medicine,* vol. xiii. p. 289.

‡ *Thérapeutique Naturelle de la Folie,* p. 76.

smiths, forgemen, moulders, &c., to engage in field labour; but it may be predicated as certain, that while, for special reasons, it may be wise to convert an engineer into a hewer of wood or a drawer of water, or to make a king trundle a barrow; that the moral effect of one inflexible rule enjoining physical labour upon all, upon expert and educated artisans, to the disregard of the healthy and habitual exercise of their natural faculties and acquired tact, would be detrimental and inoperative. But it is not perfectly clear that the *air libre* is really the panacea at Gheel which has been extolled or condemned so energetically.

In 1856, Bulckens announces that of the *tranquil* lunatics, about four-fifths were labourers, and were employed out of doors or in domestic matters. The remainder are enumerated as shoemakers, tailors, joiners, smiths, bakers, curriers, cooks, sempstresses, lace-makers, &c.,* and are very properly engaged in their original trades. He does not supply the comparative numbers, but from the table of admissions it is discovered that of 127, 49 came from towns, 78 from the country (p. 14); and that of those whose occupation could be ascertained, none of the female sand more than one half of the males had been accustomed to ply their trade out of doors.

A table furnished by M. Parigot† shows that of 204 patients employed out of 340 chargeable to Brussels, 20 worked on their own account, 40 for their guardians, who paid them 50 centimes per week, 44 assisted in the house, and 98 could do nothing more than pluck legumes.

There are several valuable principles evolved by these statistics. There is, first, the fact that about one half of the insane population is occupied; secondly, that they have access to pursuits and trades in harmony with, and calculated to exercise, their former knowledge and acquirements; and thirdly, that they have, or some of them have, a real interest in their labour, whether the money paid be regarded as an encouragement, a reward, or as wages. But what light do these figures cast upon the "air libre" system? They would appear to show that whatever advantages it may possess, it does not induce a greater number of the insane to engage spontaneously in real or even nominal activity than the moral suasion, the example, the rewards, the deprivations resorted to in asylums. Genuine spontaneity, however, appears apocryphal in the face of 50 centimes and the necessities of the guardians.

But further; it would appear that of 730 individuals returned in 1846, only 116 were agricultural servants, and enjoyed to its full extent muscular exercise in the fresh, health-bringing

---

* *Rapport*, p. 46. † *Thérapeutique Nat.*, p. 86.

breeze; that according to another account, and at another time, about one half of the active class confined their exertions to plucking beans, and that at least one-fifth (Bulckens) of the tractable, and permanently industrial because tractable class, were smiths, bakers, lacemakers, &c., who could not pursue their calling in the open air, and who are condemned to a stationary, if not to a sedentary vocation. It would not avail to press these illustrations further, but even at this stage, they point to a reduction of the claims of this system as peculiar, or distinguished from the experience of well-regulated modern asylums, in regard to the industrious class of inmates, within more moderate limits; while it leaves an undisputed superiority in the amount of freedom conceded to the idle, the excited, and those under restraint.

But, while open to what appear drawbacks, and to some insurmountable objections, when regarded as a mature institution as then in operation, or as the *sole* means by which a whole class of diseases was to be combated, Gheel contained in its arrangements the germ of further development in itself, and suggestions for modification in the existing views and practices of psychologists. The capacity for improvement supposed to be detected, was in the provision of means adapted for the treatment of cases which were obviously neglected or maltreated, while the village was preserved as the residence of others; and the example offered for imitation consisted in the country life, the domestic habits, the contact with healthy minds, and the minute classification which might be effected. My reasons for entertaining this subject, apart from Gheel, and for giving it long and anxious thought were:—1. The crowded state of asylums, and the conviction that many of their inmates might live happily and usefully under a less rigorous rule and routine than what is essential to the management of an hospital. 2. The importance which I attached to the separation of certain, and the grouping together of other classes. 3. The attainment of the home feeling, the home life, under certain limitations. 4. The belief that any arrangement is preferable to unsupervised management among strangers. 5. The impression that small groups, or families, are in keeping with the character and early habits of my countrymen. 6. The continuation of the influence of the asylum beyond the atmosphere of the asylum, and without the disturbing causes to which it is liable; and 7. a profound conviction that for many classes of the insane, irrespective of the dangerous, &c., the restraint of an asylum is salutary, and a positive source of happiness, and of greater moral and intellectual health and strength than could spring from any other mode of life. But in publishing a *vidimus* of these views it never was contemplated that the principle of asylum life should be abrogated, that every household was to be an independent society, every house a separate asylum. Even in criticising the theory, Jessen writes:—

"If, in the construction of asylums, this were taken into consideration, parts of them would be so arranged that their inhabitants could enjoy almost unlimited freedom. That the patients should be compelled to lead a regular mode of life and diet can be by no means considered an objection, especially as the permission to make any deviation from this will rest with the physician; and punctuality, even to a minute, should be provided for, &c."

The object aimed at was the incorporation of cottage residences with a central institution, of which the tenants were to be salaried officers, and in no degree dependent upon the work of their charges; from which all authority was to be delegated, all instruction, superintendence, medical and moral prescriptions were to issue; and towards which all appeals, applications, all desires for society, amusement, worship, were to gravitate. It might be matter for consideration whether the community should be surrounded by walls or other enclosures, although I conceived such an arrangement important; whether the dwellings should be solitary, or grouped together; limited to the territory specially belonging to the corporation, or that its roots and ramifications should spread into the adjoining hamlets and through the surrounding country. But it was my opinion that, whatever arrangement was adopted, the different houses should form parts of the one asylum, and be subject to the same influence and rules, or to such modifications of these as might seem advisable to the medical officers. I shall not shrink from the honour or discredit of having formulised this idea, or of having proposed its practical application as a mode of providing for and *treating* large classes of the insane. Several asylums recognising the benefits, or at all events the pleasures, of segregation and domesticity have already embraced this view, and clustered cottages around a central building. The object, however, in such instances as Devon, Aberdeen, &c., has been either to accommodate surplus numbers of chronic cases; to gratify, rather than to treat or to restore to reason; to carry out that training which is called for, and which produces such marvellous results after the chronicity and incurability of mental disease are recognised, and which is undertaken to mitigate, or elevate, or apply the characteristics of the morbid condition.

In the treatment of convalescent patients, or those discharged on trial, Dr. Bucknill anticipated or sanctioned the proposal that a part of an asylum should assume the form of a village. In the Report of the Devon County Asylum he writes:—

"A limited number of patients have been discharged on trial, and boarded with neighbouring cottagers, selected as trustworthy and amiable persons. In several instances the women of these cottages have *acquired* some experience in the right management of the insane. Some of them have been employed as occasional attendants or domestics in the asylum, and others have married asylum artisans. This experience has made them willing to accept, and qualified to undertake, the charge of such inmates of their homes. Both the patients and the persons having charge of them feel themselves under the eye of the medical superintendent, who visits them unexpectedly. The patients are extremely well satisfied and happy."*

A more close approximation to what is proposed is found in the following sentences, which appeared in the *Scotsman* newspaper, September, 1857, and are from the pen of my friend Dr. Coxe:—

"There are two ways in which the fundamental principles of the treatment followed at Gheel might be carried out. The first is, to place the patients in small houses or cottages on a large estate, under the care of paid attendants. There would in such a case be a large central house, for the reception and treatment of cases requiring special care; while the other patients would be distributed in smaller houses, according to the peculiarities of their mental affections. This plan would be a vast improvement on the present one, of gathering together all the patients in one large building . . . . . and the system of Gheel might thus be gradually and naturally introduced by removing the peaceable patients, and placing them with cottagers in the immediate neighbourhood of the parent house."

A "large estate" did not, of course, enter into my scheme. I had objected to such an arrangement as impracticable, on the ground of expense; and I regarded it as unsafe, on the ground of the distance of the members of the community from observation and treatment, and as destructive of the asylum principle. The plan, as it commended itself to my approval, did not, moreover, restrict the cottages to being depôts for dements, or homes for convalescents, although both of these classes, or individuals belonging to them, might have found shelter in them; but recognised them as parts of the general asylum, and designed them as available for various objects in classification, and during treatment, and in various forms of mental alienation. In so far, however, as Dr. Coxe's suggestion embraces an agglomeration of small houses around a central building, and that the custodiers of the insane should be paid attendants, our views are the same.

It is difficult to trace the growth of a conviction, or the elements of which it consists, but I am inclined to think that recollections of the application of a somewhat similar arrangement to the treatment of the higher classes in the establishments of Esquirol at Ivry, and Fox at Brislington, as well as my knowledge of what

* Quoted by Dr. Webster, *Journal of Psychological Medicine*, vol. x. p. 237.

Gheel did and failed to do, may have emboldened me in suggesting such a course. But my suggestion was not founded solely upon such isolated facts, but upon my own experience, and that of others, as to the extent to which the insane may safely and beneficially reside beyond the boundaries of an asylum, in offshoots or dependencies; may mingle with their fellow-men; and of the ease and rapidity with which the apprehension and antipathies of the sane as to such associations may be overcome, and the most timid familiarized with those they have been accustomed to regard as outcasts, and to endow with repulsive qualities. Some of the steps by which this result was arrived at may be illustrated by the following extracts:—

"To render this transaction gradual and safe, it is now frequently recommended, wherever affluence permits such a step, that patients who have recovered should pass a certain period, the duration of which is determined by the result of the experiment, in the residence of educated families in the vicinity of the institution. The arrangement emancipates from the stern rule of confinement without the concession of perfect liberty, from the atmosphere of the asylum surcharged with distorted views, exaggerated feelings, unbridled propensities; and affords the comforts of home and the society of healthy minds, beyond the limits, but within the moral influence of that authority to which the individual has been accustomed to yield. It revives former habits; it multiplies the tests and trials to which the restored reason must be subjected, and imparts confidence to the patient, and affords a guarantee to others of the reality and stability of the mental change; it institutes a re-education in the modes of thinking and feeling, and in the conventional amenities and graces which seclusion and protracted disease have a tendency to obliterate or impair. Many establishments of this kind have been founded and are now in operation; and, without having any connexion with the institution, may be regarded as dependencies to which the advice and authority of its officers penetrate; and which in this manner, extend the benefits of treatment to fickle and feeble capacities through varied ramifications of society, and into the centre and common walks of life. Such a course is not only probationary, but restorative. It becomes an extension of discipline deprived of its repulsive restrictions." —(*Report*, *Crichton Royal Institution for Lunatics*, 1853, p. 9.)

"Members of the community have passed the summer in the country. The connexion with the Institution has been maintained by constant visits from the officers; so that while the pursuits and pleasures of rural life poured balm into the troubled spirit, the influence and advantage of discipline were less perceptible, but not relaxed. The emancipation from monotony, which is as irksome to some minds as restraint, and the total change of impressions secured by this expedient, have not been counterbalanced by any accidents or difficulties. It must be restricted to the affluent. The west coast of Scotland is brought so close to Dumfries by railway communication, that a party enjoyed the benefits of seaside exercise as well as of change of scene.

One party paid a visit to Ireland and England, which demonstrated, at least, the practicability of such an arrangement."—(*Report, Crichton Royal Institution for Lunatics*, 1855. p. 36.)

"Patients have visited distant towns, have gathered the arbutus on the shores of Killarney, have mingled with the multitudes that crowd the Crystal Palace. Forty excursions have been made to the environs. A corps of anglers has explored every stream and many a lake in the district. Pedestrian excursions have been made to the summit of Criffell. It is confidently believed that upon no occasion has this liberty been abused; while the pursuit of game was most enthusiastically prosecuted, the sportsmen were led into scenes of great natural beauty and historic or romantic interest; were introduced into grounds and woods, and sometimes to the hospitality of the proprietors, from which they carried back impressions calculated to cheer the gloom of winter, and to displace the harsh and jealous thoughts sometimes entertained of their fellow-men."—(*Report of the Crichton Royal Institution*, 1856. p. 34.)

"Parties have resided at the seaside during the summer and autumn, received visits from their companions, and formed central points for excursions, bathing parties, and scientific expeditions. But the numbers were limited; the individuals belonged, and must continue to belong, to the affluent classes, except where indisposition demands such a change, and some special arrangement, as has happened, enables the superintendent to prescribe change of scene as a remedy. This principle has been extended. It is not merely permissible to speak of summer colonies, but of a permanent offshoot which is connected with, but is not a part of, the parent community. An ample mansion, in a well-wooded park, emulating the aspect and luxuries of a gentleman's residence, is not merely a home for convalescents, it is the portal which, to the affluent classes at least, may lead to the world of health and activity, it is a connecting link between perfect liberty and complete seclusion; it may become a scene of probation for those who have, under discipline, proved themselves capable of self-control; and where those whose temper and tendencies and habits are incompatible with the harmony and happiness of friends, or families, or communities, may find rest or exercise, as may be prescribed, and where the capacity of the one class for greater privileges, and the justifiableness of greater restraint and stringency in the other, may be tested under the most favourable circumstances. Already have the utility and benevolence of such an expedient been demonstrated. Already is this hospitable home a resort of many of the inmates, where they approach the confines of the society from which they have been excluded, without passing beyond the influence of the discipline which is to them in place of will, and self-control, and responsibility, and where for a time they lose the consciousness of that isolation, which mental disease creates, amid scenes and pursuits, and associated with minds which have all the novelty and freshness of health and freedom."—(*Report of the Crichton Institution for* 1857.

In admitting the superior qualifications of the Flemings to the race in this country in the management of the insane, the application of such principles as have been followed in Gheel is thereby limited and localised. While we must continue to look upon such an anomaly through our individual and national principles, and, it may be, prejudices, much that is claimed by such earnest and honest advocates as Bulckens, Parigot, Duval, Mundy, as to the treatment of the insane, present and potential, may be accepted; and while we continue sceptical as to the wisdom of introducing such a scheme into this or other countries, to the exclusion of every other mode of treatment, even of tried utility, we willingly receive the following as a description of the future and of a revolutionised Gheel:—

"L'arrangement et la propreté des logements, l'abondance et la bonne nourriture, le contentement des pensionaires, sont des faits constatés qui n'admettent que de rares exceptions. Les chaînes et les entraves grossières ont partout disparu pour faire place à des moyens plus humain et aussi plus efficaces. Le 'no restraint' fait journellement des progrès: les aliénés jouissent généralement de plus grand liberté. Les évasions sont peu fréquentes. Les proportions des guérisons augmente. Tout annonce enfin l'époque ou la colonie de Gheel réunira à la fois les avantages d'un régime convenable de traitement à ceux du régime de la famille et de la liberté."*

"Si nous possédions une infirmerie, nous en ferions une maison mère, un point central ou les aliénés paisibles auraient leur recours, comme cela se pratique déjà aujourd'hui à notre refuge provisoire. Nous tâcherions d'y organiser des réunions pour nos aliénés valides, des récréations, des exercices artistiques et littéraires, des instructions religieuses," &c.†

Controversy upon the size, or distribution, or even the precise destination of this centre of the new system would be idle. The erection of such a succursal building is an abandonment of the special constitution, and it may be of the special defect, which gave to Gheel prominence and popularity; and, what is of more significance, it assimilates the community to those which have already conferred such inestimable benefits upon humanity, and will augment the resources and success of the colony. The proposed fifty or sixty beds might be prudently doubled, but even these will enable the physician to practise his art; they will accommodate the aged, the infirm, the debased; they will tend to diminish, perhaps to extinguish, personal coercion, without, as is feared by Duval, as the expositor of the patriotic and conservative inhabitants, substituting imprisonment. The hospital will become what a capital is to an empire,—the seat of power, and law, and government, the source of intelligence and information, a succour to the feeble, the oppressed, and destitute.

* Duval, op. cit. p. 177.

